# Bi+ Cisgender People’s Mental Health, Smoking, and Alcohol Consumption as a Function of Their Relationship Configuration

**DOI:** 10.1007/s10508-026-03419-z

**Published:** 2026-05-25

**Authors:** Natalie Amos, Joel Anderson, Ruby Grant, Gene Lim, Adam O. Hill, Ruth McNair, Anthony Lyons, Marina Carman, Adam Bourne

**Affiliations:** 1 Australian Research Centre in Sex, Health, and Society, School of Psychology and Public Health, La Trobe University, Building NR6, Bundoora, VIC 3086 Australia; 2 Nagoya City University, School of Medicine, Nagoya, Japan; 3 General Practice and Primary Care, University of Melbourne, Melbourne, VIC Australia

**Keywords:** Bisexual, Relationships, Mental health, Smoking, Alcohol, Sexual orientation, Bisexuality

## Abstract

Multigender attracted (Bi+) people potentially face different experiences and challenges depending on the gender of their relationship partner/s. While those in same-gender relationships may be subject to anti-LGBTQA+ discrimination or abuse, those in opposite-gender relationships may experience greater distress associated with the invisibility or erasure of their sexual identities. These experiences may in turn impact the mental health of Bi+ people who frequently report worse mental health outcomes than their gay or lesbian peers. Data from 1261 bisexual and pansexual cisgender adults in Australia were used to explore their mental health and well-being outcomes as a function of their current relationship configuration (opposite gender partner, same gender partner, or single). Relationship configuration was found to be associated with mental health and well-being outcomes. This was especially the case for Bi+ women, who reported worse mental health outcomes if they were single or in an opposite-gender relationship. While the effect was less pronounced for Bi+ men, those who were in a same-gender relationship were less likely to have a history of smoking. The findings highlight the need for supporting the mental health and well-being of Bi+ cisgender people, which may include promoting awareness and inclusion of Bi+ identities within both the broader and LGBTQA+ communities. The findings may also have implications for developing targeted interventions aimed at reducing substance use among Bi+ individuals, particularly when single.

## Introduction

Bi+ people are those who are attracted to more than one gender (multigender attracted) and may use terms such as bisexual, pansexual, or queer. They likely make up a large proportion of the LGBTQA+ population in Australia (Hill et al., 2020, 2021; Richters et al., 2014) and frequently report worse mental health outcomes than their monosexual (attracted to only one gender, i.e., heterosexual, gay, or lesbian) peers (Hill et al., 2020; McGorray et al., 2024; Ross et al., 2018; Salway et al., 2019). Findings from previous research both globally and in Australia suggest higher rates of mental-ill health, suicidality, and self-harm compared to heterosexual and gay or lesbian counterparts (Batchelder et al., 2021; Lyons et al., 2022; Salway et al., 2019; Swannell et al., 2016). Bi+ people have also been found to report higher rates of homelessness, poverty (McNair et al., 2017; Ross et al., 2016), and violence, including sexual assault and violence from an intimate partner (Hill et al., 2020). Poor mental health and well-being outcomes are likely to be multifaceted but may be attributed to experiences of biphobia (the fear, hatred, or intolerance of Bi+ people), bi-invisibility or bi-erasure (the denial or dismissal of Bi+ identities), and historical monosexism in society (e.g., Angelides, 2001; McGorray et al., 2024; Ross et al., 2010). However, the experiences of Bi+ people and resulting mental health outcomes are frequently overlooked or are subsumed within broader LGBTQA+ research where the experiences of this population are not explored independently.

Bi+ people are often subject to considerable prejudice within both sexual minority and heterosexual (Anderson & Maugeri, 2024; Feinstein et al., 2021; Roberts et al., 2015) communities. Negative stereotypes around bisexuality impact monosexual people’s attitudes to dating or intimate involvement with a bisexual partner, with Bi+ people experiencing high rates of intimate discrimination (Armstrong & Reissing, 2014; Gleason et al., 2018). For example, previous research found that heterosexual women expressed high levels of insecurity, worry, pressure, and jealousy toward relationships with bisexual men (Armstrong & Reissing, 2014). Comparatively, heterosexual men have been found to hypersexualise bisexual women, in the form of increased sexual interest, requests for threesomes, and inquiries about their sexual history (DeCapua, 2017). Stereotypes about Bi+ individuals are also common within LGBTQA+ communities, where Bi+ individuals are often unfairly presumed to be experimenting and will invariably settle into a life of heterosexuality (Lim & Hewitt, 2018). According to several studies, both heterosexual and lesbian/gay individuals are less willing to romantically or sexually engage with bisexual partners compared to other bisexual individuals (Breno & Galupo, 2008; Feinstein et al., 2014).

This is compounded by experiences of bi-invisibility or bi-erasure e (e.g., McGorray et al., 2024; Ross et al., 2010). Many Bi+ people experience challenges expressing or communicating their identity to others (Davila et al., 2019; Hayfield, 2013; Nelson, 2020), and consequently report feeling that their sexual identities are invisible (McGorray et al., 2024; Ross et al., 2010). These experiences are thought to denote a lack of access to identity-affirming experiences, and so are associated with poor mental health and well-being outcomes for Bi+ people (Feinstein et al., 2019; Garr-Schultz & Gardner, 2021; Maimon et al., 2021; McGorray et al., 2024; Ross et al., 2018). Bi+ people may further feel that their identity is made invisible when in a romantic relationship that is perceived to be heterosexual (Bostwick & Hequembourg, 2014; Flanders et al., 2015, 2016; Garr-Schultz & Gardner, 2021; McGorray et al., 2024). Consequently, the orientation of their relationships (whether with a same- or opposite-gender partner) could potentially impact mental health outcomes. While those in opposite-gender relationships may experience greater distress associated with the invisibility or erasure of their sexual identities and potential loss of connection to LGBTQA+ community, those in same-gender relationships may be subject to anti-LGBTQA+ discrimination or abuse due to the visibility of their sexual orientation. Relationships are, therefore, an important focal point for exploring the well-being of Bi+ people.

Different challenges are encountered by Bi+ people who are single. Those who are single may not face the same challenges of discrimination as those in a same-gender relationship and may not feel that their identity is made invisible by being in a perceived heterosexual relationship. However, singlehood may be associated with its own challenges of invisibility and those who are single may miss out on the potential protective benefits of being in a committed relationship (Feinstein et al., 2016; Lyons et al., 2022).

While research on the impacts of partner gender and sexuality on bisexual people’s mental well-being has been limited, several studies have begun to explore these nuances, with mixed results. Most of this research focuses on bisexual women’s differing experiences based on partner gender. For example, one recent study found that bisexual women in monogamous relationships with lesbian-identified partners reported lower stress levels than those with heterosexual partners (Vencill et al., 2018). Furthermore, previous research has found that bisexual women in relationships with heterosexual men were less likely to be open about their bisexuality, which is often associated with poorer mental health (Xavier Hall et al., 2021). Additionally, research has found that not only can bi-negativity impact bisexual people’s mental health and sexual and relationship satisfaction, but these also can impact their partner’s satisfaction. Specifically, Mark et al. (2020) found that the perceived illegitimacy of bisexuality was negatively associated with both partners’ sexual and relationship satisfaction. Research to-date has yet to specifically explore the mental health of Bi+ people in a relationship with a Bi+ partner.

The present study uses data from the largest national survey of LGBTQA+ adults to date in Australia to explore the mental health and well-being of Bi+ cisgender women and men as a factor of their relationship configuration, whether single, in a same-gender relationship or in an opposite-gender relationship. We pose that Bi+ people in a same-gender (i.e., queer-presenting) relationship will have different social experiences as a function of their partner’s gender than those in an opposite gender (i.e., hetero-presenting) relationship. These differences include both protective factors and risk factors—for instance, those in queer presenting relationships might have greater access to queer communities and related social support but also heightened risk of experiencing prejudice, while those in hetero-presenting relationships might be more protected from sexual prejudice but risk the effects of bi-erasure. As such, the major aims of the study seek to explore whether relationship configuration is associated with mental health and well-being outcomes, as well as whether this differs between Bi+ cisgender women and men.

The study presented focuses specifically on cisgender people. We arrived at this decision for several reasons. First, trans and gender diverse people face unique and additional challenges, such as increased exposure to hostility and violence (Hill et al., 2020) compared to cisgender people, which may confound outcomes without a specific and nuanced exploration of their experiences. Second, the intersections of plurisexuality and gender diversity in relationships require a more nuanced exploration than we can provide, which warrant their own focussed investigation.

## Method

### Participants and Procedure

Data were drawn from the *Private Lives 3* national survey of LGBTQ+ adults aged 18 and older in Australia. The *Private Lives 3* survey was open to participation from July to October 2019, and participants were recruited through targeted social media advertising on Facebook and Instagram, and promotion through the networks of Australian LGBTQ+ community organizations. The survey covered a broad range of health and well-being outcomes for the LGBTQA+ community in Australia and was designed in close consultation with a Community Advisory Board. Several approaches were used to prevent or detect fraudulent responses to the survey. First, participation in the survey was voluntary and no compensation was offered for completion. The survey was hosted through the online platform Qualtrics, which uses cookies to prevent individuals from submitting multiple responses from the same IP address. Additionally, the *Private Lives 3* investigator team carefully checked through the data and removed responses that appeared likely to be false. This included carefully checking responses from participants who were over the age of 80 years old or who scored extremes on standardized scales, such as a score of 50/50 or 10/50 on the K10. It also included reviewing responses to open-text questions, particularly gender identity and sexual orientation questions, and excluding survey responses that appeared intentionally malicious. In total, 2,490 incomplete responses were removed from the final dataset as well as seven complete but presumed fraudulent or bad faith responses.

The analyses for the present paper included only respondents whose gender identity aligned with the sex that they were assigned at birth (i.e., “cisgender”), as (Hill et al., 2020) described above. Similarly, only participants who were single or indicated that their current partner/s were only cisgender men or only cisgender women were included to ensure that the current relationship configurations of respondents were ones that may be perceived or experienced as hetero-presenting compared to one that may be perceived or experienced as queer-presenting, in line with our arguments above. In summary, in order to test our hypotheses, we opted to use only cisgender participants—however, we acknowledge that Bi+ trans people’s experiences of relationships are more likely to have additional factors that shape their experiences than those of Bi+ cisgender people, and as such these relationships warrant a focused exploration that we are unable to provide with this dataset. The final study sample included 1,261 cisgender Bi+ participants.

### Measures

*Sexual orientation and relationship configuration* To identify participants’ sexual orientation, participants were first asked to choose all relevant identity labels from 12 options: “gay”, “lesbian”, “bisexual”, “pansexual”, “queer”, “asexual”, “homosexual”, “heterosexual”, “prefer not to answer”, “prefer not to have a label”, “don’t know”, and “something different.” Participants who selected more than one identity label were then asked to choose one label they identified with the most. For analysis in the present study, only participants who identified as bisexual or pansexual were included. Queer participants (n = 337 who met the other inclusion criteria) were not included in the study sample as it is unclear from the data collected whether those who identified as queer were single-gender or multigender attracted.

Participants who indicated that they were currently in a committed relationship/s were further asked to describe the gender of their partner/s, with a list of seven options to choose from, including the options not to say or to specify a gender that was not listed. Only those who indicated that their partner/s were only cisgender men or women (i.e., they did not select both cisgender men and women) were included in the analyses. This approach was taken to ensure that the current relationship configuration of respondents, whether they had one or multiple partners, was one of only opposite- or same-gender relationship/s.

Responses to the committed relationship/s item and partner/s gender item were used to code a new variable indicating if participants were single, in a same-gender relationship/s or in an opposite-gender relationship/s.

*Psychological distress* Levels of psychological distress were measured using the 10-item Kessler Psychological Distress Scale (K10) (Kessler et al., 2002). The K10 asks participants to respond to 10 items regarding symptoms of anxiety or depression that participants may have experienced over the previous 4 weeks on a 5-point Likert-type scale, ranging from “*None of the time*” to “*All of the time*.” Scale scores range from 10 to 50, with scores of 22 or more indicating high or very high level of psychological distress (Australian Bureau of Statistics, 2017). The K10 was found to have adequate internal reliability in this study sample (Cronbach’s a = 0.92).

*Anxiety and depression:* Anxiety and depression were assessed by asking participants “In the past 12 months, have you been treated for or diagnosed with any of the following?” Participants were asked to select all that applied to them from a list of ten mental health diagnoses, such as “Depression” and “Generalized Anxiety Disorder” and the additional option to specify a condition that was not listed, or to select “none of the above.” Dichotomous variables were generated to identify whether or not participants had been diagnosed or treated for depression, and whether or not they had been diagnosed or treated for generalized anxiety disorder in the past 12 months (1 = Yes; 0 = No).

*Suicidality* Recent experiences of suicidal ideation were examined by asking participants if they had experienced any thoughts related to suicide or wanting to end their life. Response options included “*No*,” “*Yes, in the past 12 months*,” “*Yes, more than 12 months ago*,” and “*Prefer not to answer.*” Participants were able to select multiple responses if they did not choose “*No*” or “*Prefer not to answer*.” In order to explore recent experiences, binary variables were generated indicating whether or not participants had experienced suicidal ideation in the past 12 months.

*Alcohol struggle* To assess difficulties with alcohol use, participants were asked ‘In the past 12 months have you experienced a time where you have struggled to manage your alcohol use or where it has negatively impacted your everyday life?’ Response options were “*Yes*” or “*No*.”

*Smoking* Participants’ smoking history was assessed by asking participants if they smoke cigarettes or any other tobacco products. Participants were able to indicate if they had “*never smoked*”, had “*previously smoked*” or “*currently smoke*.” A dichotomous variable was generated to identify those with and without a history of smoking.

### Statistical Analyses

All analyses were performed using Stata (Version 16.1, StataCorp, College Station, TX, USA). Multivariable logistic regression analyses were conducted to assess the relationship between relationship configuration and each of the outcome variables, as described in the measures, while controlling for the confounding effects of age and residential location. These logistic regressions were repeated for cisgender Bi+ women and cisgender Bi+ men. While both groups contain large enough sample sizes for adequate power, direct comparisons between women and men were not conducted due to the substantial imbalance of sample sizes (1:4) as well as markedly different baseline outcomes between these two groups (see Table 2). Conducting multivariable logistic regression analyses for each gender, allows for meaningful interpretation within each gender group, while avoiding the confounding effects of differences between women and men in baseline characteristics. All multivariable regression analyses were conducted with ‘single’ as the reference category and then repeated with ‘opposite-gender relationship’ as the reference category, in order to directly compare opposite and same-gender relationships. Results are reported as adjusted odds ratios (AOR) with 95% confidence intervals (CIs) and *p* < 0.05 used to determine statistical significance.

## Results

Table 1 presents the characteristics of the study sample. Of the total sample of 1,261 participants, 252 were cisgender men and 1,009 were cisgender women. The mean age of participants was 28.3 years (*SD* = 11.0). One thousand and twenty-two (81.1%) participants identified as bisexual and 239 (19.0%) as pansexual. Approximately half of the men (50.8%; *n* = 128) and just under half of the women (45.9%; n = 463) were single (46.9% of total sample; *n* = 591). One-third of men (33.3%; *n* = 84) and just over one-third of women (38.9%; *n* = 392) were in an opposite-gender relationship (37.8% of total sample; *n* = 476). Smaller proportions of men (15.9%; *n* = 40) and women (15.3%; *n* = 154) reported being in a same-gender relationship (15.4% of total sample; *n* = 194).

**Table 1:** Sample characteristics

	n	%
*Gender*		
Cisgender man	252	20.0
Cisgender woman	1009	80.0
*Sexual orientation*		
Bisexual	1022	81.0
Pansexual	239	19.0
*Relationship configuration*		
Single	591	46.9
Opposite gender	476	37.7
Same gender	194	15.4
*Residential location*		
Capital city, inner suburban	464	37.2
Capital city, outer suburban	386	30.9
Regional city or town	322	25.8
Rural/Remote	76	6.1
*Ethnic background*		
White European	985	87.6
Asian	36	3.2
Multi-racial	94	8.4
Something else	10	0.9

	M	SD
Age (in years)	28.3	11.0

**Fig. 1:**
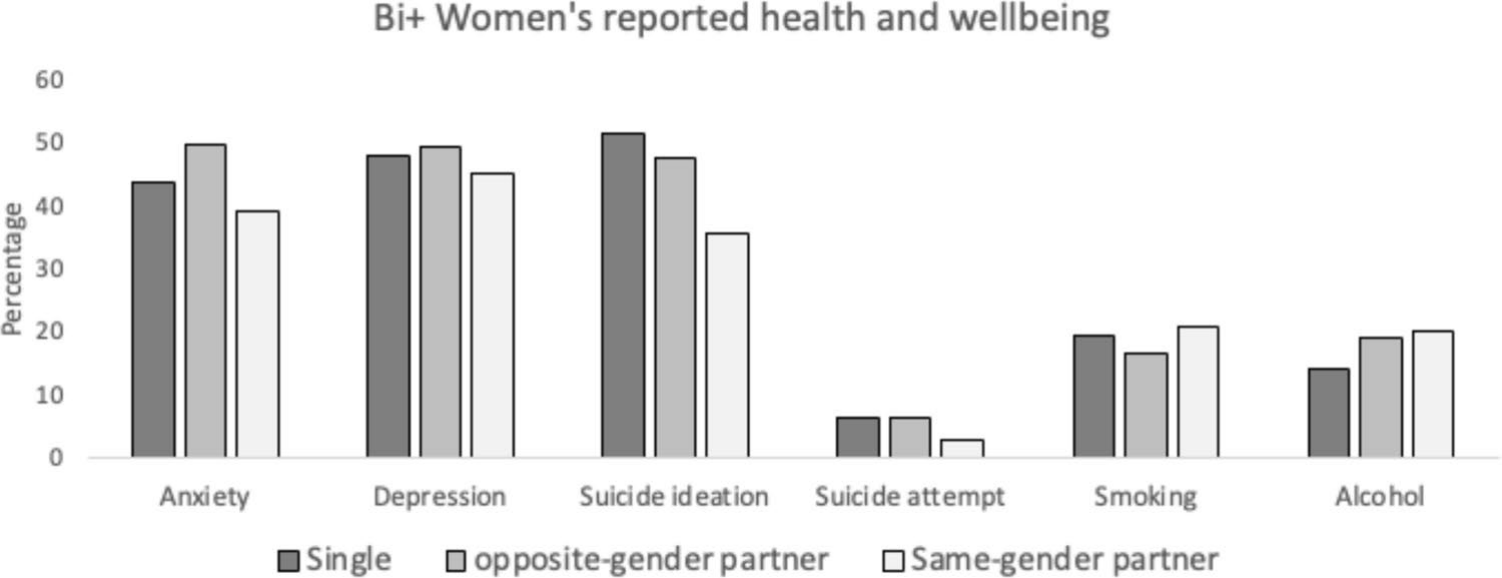
Reported frequency of categorical health and well-being variables for Bi+ women as a function of participants’ relationship configuration

**Fig. 2:**
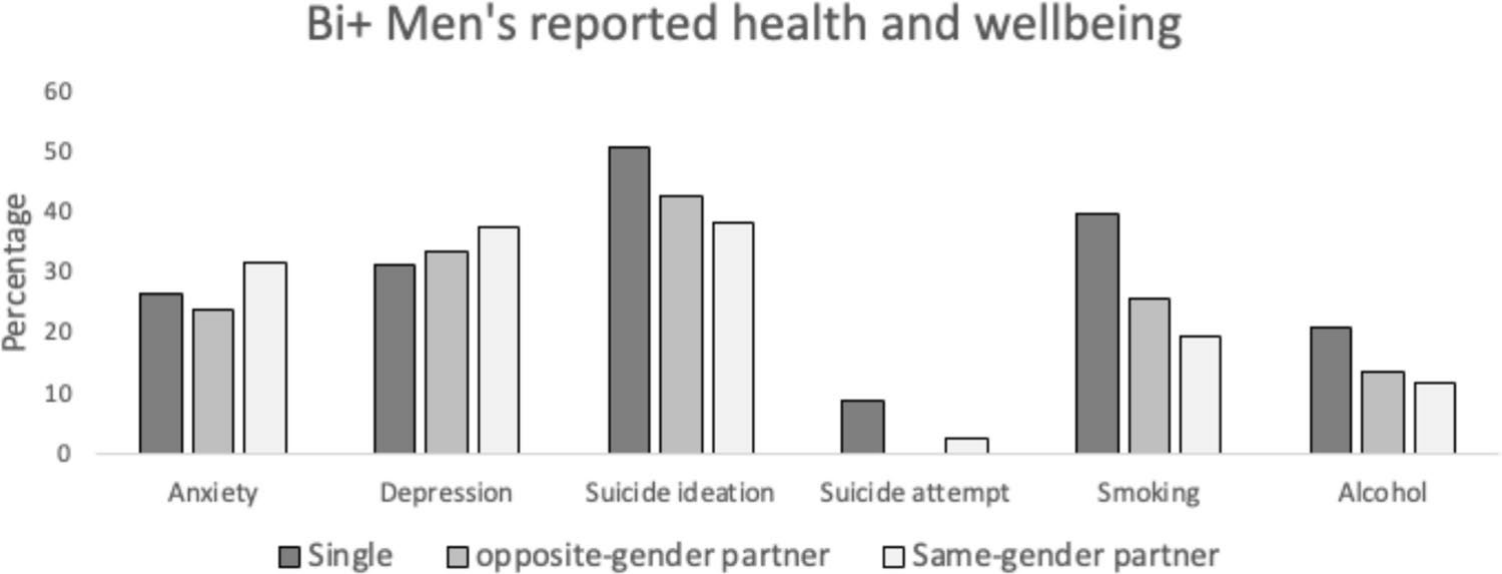
Reported frequency of categorical health and well-being variables for Bi+ men as a function of participants’ relationship configuration

The frequencies (and percentage of valid cases) of reporting each outcome variable are presented in Table 2, and these are also presented visually in Fig. 1 for Bi+ women and Fig. 2 for Bi+ men.

**Table 2:** Multivariable logistic regression results for cisgender men and women

	Cisgender men (n = 252)				Cisgender women (n = 1,009)			
	n	%	AOR (95% CI)	*p*-value	n	%	AOR (95% CI)	*p*-value
Psychological Distress (K10 High/Very High)								
Single	67	53.2	REF		343	75.6	REF	
Opposite-gender relationship	40	48.8	1.04 (0.56–1.91)	0.909	269	70.6	0.87 (0.63–1.20)	0.389
Same-gender relationship	21	55.3	1.21 (0.56–2.60)	0.635	82	54.3	0.54 (0.36–0.81)	**0.003**
Same vs opposite-gender relationship	–	–	*0.86 (0.38–1.93)*	*0.714*	–	–	*0.62 (0.42–0.93)*	***0.020***
Depression (12 months)								
Single	37	30.8	REF		213	47.3	REF	
Opposite-gender relationship	30	36.1	1.37 (0.72–2.64)	0.339	194	50.4	1.13 (0.85–1.49)	0.396
Same-gender relationship	15	37.5	1.41 (0.62–3.21)	0.415	65	46.1	1.02 (0.69–1.50)	0.939
Same vs opposite-gender relationship	–	–	*0.98 (0.43–2.20)*	*0.951*			*0.90 (0.61–1.34)*	*0.602*
Anxiety (12 months)								
Single	30	25.0	REF		197	43.8	REF	
Opposite-gender relationship	18	21.7	0.82 (0.38–1.76)	0.614	194	50.4	1.47 (1.11–1.95)	**0.008**
Same-gender relationship	14	35.0	1.58 (0.67–3.72)	0.290	57	40.4	1.13 (0.76–1.69)	0.537
Same vs opposite-gender relationship	–	–	*0.63 (0.24–1.62)*	*0.333*	–	–	*0.77 (0.52–1.15)*	*0.205*
Recent suicidal ideation								
Single	62	49.6	REF		227	50.3	REF	
Opposite-gender relationship	35	42.2	0.89 (0.48–1.63)	0.706	181	47.6	0.95 (0.71–1.25)	0.703
Same-gender relationship	13	33.3	0.51 (0.23–1.16)	0.110	51	33.6	0.61 (0.41–0.90)	**0.013**
Same vs opposite-gender relationship	–	–	*0.51 (0.22–1.20)*	*0.123*	–	–	*0.64 (0.43–0.95)*	***0.028***
Alcohol struggles								
Single	21	17.8	REF		55	13.7	REF	
Opposite-gender relationship	9	11.8	0.58 (0.24–1.41)	0.232	68	19.1	1.48 (1.00–2.19)	0.051
Same-gender relationship	3	8.8	0.38 (0.10–1.49)	0.165	26	19.1	1.42 (0.84–2.40)	0.195
Same vs opposite-gender relationship	–	–	*0.38 (0.10–1.49)*	*0.165*	–	–	*0.96 (0.56–1.60)*	*0.875*
Smoking (Any)								
Single	47	36.7	REF		89	19.3	REF	
Opposite-gender relationship	21	25.0	0.62 (0.32–1.20)	0.155	68	17.3	0.87 (0.61–1.24)	0.440
Same-gender relationship	7	17.5	0.37 (0.14–0.95)	**0.039**	29	18.8	0.95 (0.59–1.55)	0.847
Same vs opposite-gender relationship	–	–	*0.37 (0.13–1.02)*	*0.055*	–	–	*1.10 (0.67–1.79)*	*0.713*

n’s and %’s represent the frequency and proportion of participants in each category who responded affirmatively to the outcome variable; italicised rows present the results from additional regression analyses with opposite-gender relationships as the reference category

### Mental Health and Suicidality

Table 2 presents the multivariable logistic regression results for both cisgender Bi+ men and women for all outcomes.

Among cisgender Bi+ men, no statistically significant associations were found between relationship configuration and psychological distress, depression, anxiety or suicidal ideation.

Results demonstrated a different pattern among cisgender Bi+ women. Bi+ women in same-gender relationships had lower odds of reporting high or very high psychological distress (AOR = 0.54, CI = 0.36–0.81, *p* = .003) and lower odds of reporting recent suicidal ideation (AOR = 0.61, CI = 0.41–0.9, *p* = .013) compared to those who were single. Those in opposite-gender relationships had higher odds of reporting anxiety in the past 12 months compared to single women (AOR = 1.47, CI = 1.11–1.95, *p.* = .008). Additionally, Bi+ women in same-gender relationships had significantly lower odds of reporting high/very high psychological distress (AOR = 0.62, CI = 0.42–0.93, *p* = 0.020) and recent suicidal ideation (AOR = 0.64, CI = 0.43–0.95, *p* = 0.028) compared to those in opposite-gender relationships.

### Smoking and Alcohol Struggle

Among cisgender Bi+ men, relationship configuration was not associated with alcohol struggle. However, Bi+ men in same-gender relationships had significantly lower odds of smoking compared to single men (AOR = 0.37, CI = 0.14–0.95, *p* = .039).

No significant associations were found between relationship configuration and smoking or alcohol struggle among cisgender Bi+ women. However, the association with alcohol struggle approached significance (AOR = 1.48, CI = 0.998–2.19, *p* = .051).

## Discussion

The mental health and well-being of Bi+ people is frequently found to be worse than that of their lesbian, gay or heterosexual peers (Hill et al., 2020; McGorray et al., 2024; Ross et al., 2018; Salway et al., 2019). However, limited research has explored the various contributors to this disparity in well-being outcomes for Bi+ people. Present findings provide evidence that relationship configuration may be a contributing factor to the mental health differences observed in the literature for cisgender Bi+ people. In particular, relationship configuration (single, opposite-gender, or same-gender relationship) was found to be associated with mental health and well-being outcomes for cisgender women, who reported worse outcomes if they were single or in an opposite-gender relationship. While fewer associations were found for Bi+ cisgender men, relationship configuration was found to be associated with a history of smoking.

### Mental Health and Suicidality

Mental health and suicidality were not associated with relationship configuration for Bi+ men, however, a number of outcomes were found to differ for Bi+ women. Specifically, Bi+ women in same-gender relationships reported the lowest rate of reporting high or very high psychological distress and suicidal ideation in the past 12 months, while those in opposite-gender relationships were more likely than single Bi+ women to report a diagnosis or treatment for anxiety in the past 12 months. Notably, for Bi+ cisgender men, no associations were found between relationship configuration and mental health or suicidality outcomes.

These differences observed between Bi+ women and men suggest the differential impact that relationship configuration may have on their well-being. The more pronounced mental health challenges faced by Bi+ women in opposite-gender relationships may be attributed to several factors. Bi+ women, as with heterosexual women, in relationships with men may be disadvantaged by heteronormative relationship dynamics—such as those resulting in unfair divisions of labor within the home. In contrast, cisgender men, regardless of partner gender may be protected by a form of male privilege that they are afforded within their relationships, such as benefiting from an unfair division of labor. Additionally, Bi+ women are more likely to experience fetishization by opposite-gender partners, a phenomenon that can lead to a range of negative emotional and psychological outcomes. For example, DeCapua (2017) found that bisexual women face pervasive fetishization from heterosexual men. This experience of objectification may, in turn, result in worse mental well-being outcomes (Sáez et al., 2019). It is also possible that Bi+ women have higher identity centrality than Bi+ men, meaning that their identity may be more important to them (Ashmore et al., 2004; Hinton et al., 2022). Higher identity centrality may lead to greater efforts to make their identity visible (Davila et al., 2019; Feinstein et al., 2021), and a stronger association between the visibility of their identity and mental well-being (McGorray et al., 2024). However, these explanations are speculative and further research of Bi+ people is necessary to better understand the gender differences observed in the present study.

Mental health outcomes as a factor of relationship configuration among Bi+ women may relate to their experience of visibility or erasure of their identity within their relationships, and their subsequent engagement with the LGBTQA+ community. Experiences of rejection from both heterosexual and LGBTQA+ communities (Feinstein et al., 2021; Roberts et al., 2015), as well as experiences of bi-erasure and the invisibility of their sexual identity have been found in previous research to have a negative impact on the well-being of Bi+ people (e.g., McGorray et al., 2024; Ross et al., 2010). Conversely, feeling connected to the LGBTQA+ community has been found to be associated with lower psychological distress among sexual minority women who felt that this community connection was positive for them (Lim et al., 2023). Moreover, a recent daily diary study of Bi+ people found that participants’ attempts to make their identity visible, while making them more vulnerable to anti-bisexual experiences, were associated with more positive well-being outcomes (Dyar et al., 2022). While speculative, it is possible that Bi+ women in opposite-gender relationships experience poorer mental health outcomes due to the erasure of their identity or lack of connection to LGBTQA+ community. It is of note that this association was only observed for Bi+ women in the present study, and not for men, which perhaps suggests a greater negative impact of the experience of bi-erasure or invisibility for women than men.

While more research is required to better understand the nuance of these outcomes, the findings highlight that both the visibility of Bi+ women’s identities and the configuration of their relationships are important for understanding and supporting their mental health and well-being. This may have particular implications within support and healthcare settings, where assumptions about identity based on the gender of their partner may contribute to identity erasure or misunderstanding of their needs.

### Smoking and Alcohol

Associations with substance use also differed somewhat for Bi+ women and men. Bi+ women reported no associations between relationship configuration and alcohol struggle or smoking. Bi+ men similarly showed no association between relationship configuration and alcohol struggle. However, Bi+ men who were in a same-gender relationship were the least likely to report a history of smoking than those who were single. Notably, no significant differences were found between men in same and opposite gender relationships.

For Bi+ men, the higher incidence of smoking among singles could suggest that single men may engage in smoking due to social factors (Smith et al., 2018) or as a stress response (Gordon et al., 2021). Previous research also suggests that dating prospects may be a motivation to quit smoking (Matthews et al., 2017), resulting in less smoking among those in a relationship. However, the interpretation of these findings is limited by the time frames of the variables used (i.e., history of smoking; current relationship configuration), and further research is necessary to better understand this association. It is possible that current relationship dynamics reflect past partner influences or vice versa. Although our analysis is correlational and does not establish causality, it does highlight the possibility that singlehood may be a risk indicator for substance use. This could have implications for developing targeted interventions aimed at reducing substance use among Bi+ individuals, particularly when single.

### Limitations

The sample is likely to be limited by self-selection bias, and it is possible that the Bi+ people who participated in this survey have higher identity centrality. Previous research has illustrated that Bi+ people with higher identity centrality are more likely to actively engage in efforts to make their identity visible (Davila et al., 2019; Feinstein et al., 2021), suggesting that the visibility of their identity may be of greater importance to them and may have a stronger association with their mental well-being (McGorray et al., 2024). Those who engaged with the *Private Lives 3* survey may have done so because they have higher identity centrality and therefore the findings from the study may be more pronounced in this sample.

Additionally, the cross-sectional nature of our survey limits our ability to draw conclusions about the temporality or causality of the observed associations. Participants’ relationship configuration was in reference to their current relationship partner gender, while some of the outcome variables were retrospective, such as past 12-month alcohol struggle. This discrepancy poses challenges in understanding the sequence of independent and dependent variables.

The survey also did not capture whether participants had disclosed their sexual orientation to their relationship partner, or whether their partners were accepting of their identities. This is likely to be an important factor to consider for the well-being of those in opposite gender relationships. Furthermore, without comprehensive relationship histories, we cannot ascertain the full impact of past relationships on current well-being. For instance, a Bi+ woman currently in an opposite-gender relationship who has a history of exclusively dating opposite-gender partners may experience bi-erasure more acutely than someone with a more diverse relationship history. This underlines the importance of longitudinal research designs in future studies to unravel the dynamics between relationship configuration history and mental health outcomes in the Bi+ population.

Finally, while this paper aimed to understand the impact of being in a relationship that may be perceived by others as heterosexual compared to a same-gender relationship for cisgender Bi+ people, further research is necessary to explore the experiences of trans and gender diverse people who identify as Bi+. It would be of interest and a valuable contribution to the literature to conduct similar and more nuanced analyses with trans and gender diverse people to understand their experiences within difference relationship configurations. This work could also extend to exploring those in relationships with people of all genders, rather than just focusing on people with cisgender partners.

### Conclusions

The findings demonstrate variance in mental health and well-being among Bi+ cisgender people as a factor of their relationship configuration. Most notably, Bi+ women in opposite-gender relationships (those that may be perceived to be heterosexual relationships) have significantly worse mental health than women in same-gender relationships. These findings highlight the importance of a nuanced approach to understanding and addressing the experiences of Bi+ cisgender individuals, recognizing that their needs and experiences can vary significantly depending on factors like gender and relationship configuration. This may be especially important within healthcare settings. Health providers must be willing to explore current relationship and sexual orientation of their patients and not assume all people in opposite-gender relationships are heterosexual. The findings also highlight the need to develop awareness and inclusion of Bi+ people within LGBTQA+ communities regardless of their relationship status or configuration.

## Data Availability

The data used for this study have not been made publicly available.
